# Evaluating tools for predicting and measuring radiometric performance of germicidal ultraviolet systems

**DOI:** 10.1111/php.70080

**Published:** 2026-02-18

**Authors:** Valeria Holland, Belal Abboushi, Eduardo Rodriguez‐Feo Bermudez, Jason Tuenge, Annabelle Johnson, Shohoria Shorno, Ernest R. Blatchley, Gabe Arnold

**Affiliations:** ^1^ Pacific Northwest National Laboratory Richland Washington USA; ^2^ Purdue University West Lafayette Indiana USA

**Keywords:** fluence rate, germicidal ultraviolet, GUV, irradiance, UV‐C

## Abstract

Germicidal ultraviolet (GUV) air treatment technologies can be effective and safe for reducing airborne disease transmission. Today, GUV systems are designed and evaluated using simulation and measurement tools that require further assessment of their accuracy in estimating fluence rate and irradiance. This article reports results from two experiments where two simulation software (Visual Lighting and Photopia) and two measurement techniques (tetrahedron and cubic approximations) were evaluated against chemical actinometry for quantification of GUV fluence rate in a chamber. Additionally, Visual and Photopia were compared to measurements of planar UV‐C irradiance for eye and skin exposure. Results showed that overall mean fluence rates were similar between actinometry and both simulation software for WR GUV systems as well as between actinometry and Photopia for UR GUV systems. The tetrahedron approximation better predicted overall mean fluence rate for WR and UR GUV systems, compared to a cubic approximation which tended to overestimate it. Compared to measurements, simulated eye and skin irradiance varied, with higher variability in simulated eye irradiance. The evaluated simulation software can be used to guide the design of GUV systems but must be supplemented with in situ measurements.

## Graphical abstract

An evaluation of simulation and measurement tools for germicidal ultraviolet (GUV) systems is presented to inform their design and deployment to control the spread of airborne pathogens.

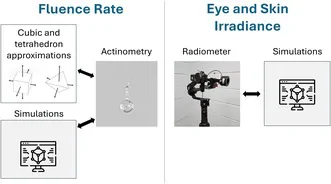

**Abbreviations**
ACGIH: American Conference of Governmental Industrial Hygienists
FOV: field of view
GUV: germicidal ultraviolet
IES: illuminating engineering society
IQR: interquartile range
KrCl*: Krypton‐Chloride Excimer (lamp type)
LED: light emitting diode
LLF: light loss factor
LPM: low‐pressure mercury (lamp type)
MRBE: median relative bias error
RBE: relative bias error
RMSE: root mean square error
UR: upper‐room (GUV system type)
UV‐C: ultraviolet‐C (wavelength range 20,0280 nm)
WR: whole‐room (GUV system type)

## INTRODUCTION

In‐room germicidal ultraviolet (GUV) air treatment systems offer potential health and safety benefits by reducing the risk of airborne disease transmission in buildings. GUV systems have been used in healthcare and other high‐risk settings to control the spread of tuberculosis and other airborne pathogens for decades.^1^, ^2^, ^3^ There has been renewed interest in GUV since the start of the COVID‐19 pandemic, and it has been shown to be effective at inactivating the SARS‐CoV‐2 virus.^4^ GUV technologies have also been shown to be an energy‐efficient option to reduce pathogen concentration in the air.^5^

There are two common types of in‐room GUV systems that can be used in occupied spaces: upper‐room (UR) and whole‐room (WR) systems. As their names indicate, these two types irradiate different portions of a room, and they do so using different UV‐C (200–280 nm) wavelengths. UR systems typically use low‐pressure mercury (LPM) lamps with peak radiant output at 254 nm or UV LEDs with a peak in the range 260–280 nm. WR systems utilize far UV‐C radiation with a peak at 222 nm, with the most common source being optically filtered krypton‐chloride excimer (KrCl*) lamps.

There are two main considerations for designing GUV systems: effectiveness and safety. The effectiveness of GUV to inactivate pathogens depends on various factors, such as the GUV dose—which is dependent on the spatial distribution and amount of radiant energy, fluid mechanics, and air mixing—the UV spectral distribution, and the targeted pathogen(s). UV dose is defined as the time‐integral of fluence rate (total radiant power per unit cross‐sectional area).

Besides ensuring effectiveness, GUV systems must be designed to comply with safety standards for UV exposure that determine safe planar UV irradiance limits for human eyes and skin based on the spectrum of the UV source. Note that simulations typically do not account for occupant's motion and movement in space, which affect UV exposure, hence care should be taken when interpreting the relevance of simulated irradiance values to exposure limits.

Currently, GUV systems are designed and evaluated using simulation tools and measurement techniques adapted from the architectural lighting industry. For UV‐C wavelengths, there is a need for more studies to assess the accuracy of these tools and techniques.

In a previous study, simulations of UR installations were found predictive of average fluence rate but had poor prediction of fluence rate at individual points.^6^ This study did not assess photobiological safety predictions. Other studies on GUV compared field measurements with simulated values for UV irradiance but had various limitations. For example, some studies did not consider room surface reflections,^7^, ^8^, ^9^, ^10^, ^11^ did not provide direct comparisons between simulated and measured values,^12^ used simulations or measurements by others in lieu of collecting measurements,^13^ or only considered UV‐A.^14^

Regarding in situ UV measurements, there is currently no industry standard for measuring fluence rate in the field to ensure that the installed GUV system performs as intended. Spherical actinometry is used for measuring fluence rate, but it is generally limited to laboratory settings since it involves using chemicals and fragile spherical quartz containers.^15^, ^16^, ^17^ Rudnick et al. used a cylindrical sensor and planar irradiance to measure fluence rate in the field.^6^ They found that cylindrical irradiance had the worst agreement with simulations, compared to irradiance and actinometry measurements. Cylindrical irradiance is by definition different from fluence rate. Planar irradiance is only a good approximation of fluence rate if there is only one active luminaire in the space, room surfaces have essentially zero reflectance, and the sensor is aimed at the center of the luminaire; such a scenario may be unrealistic in practice.

**FIGURE 1:**
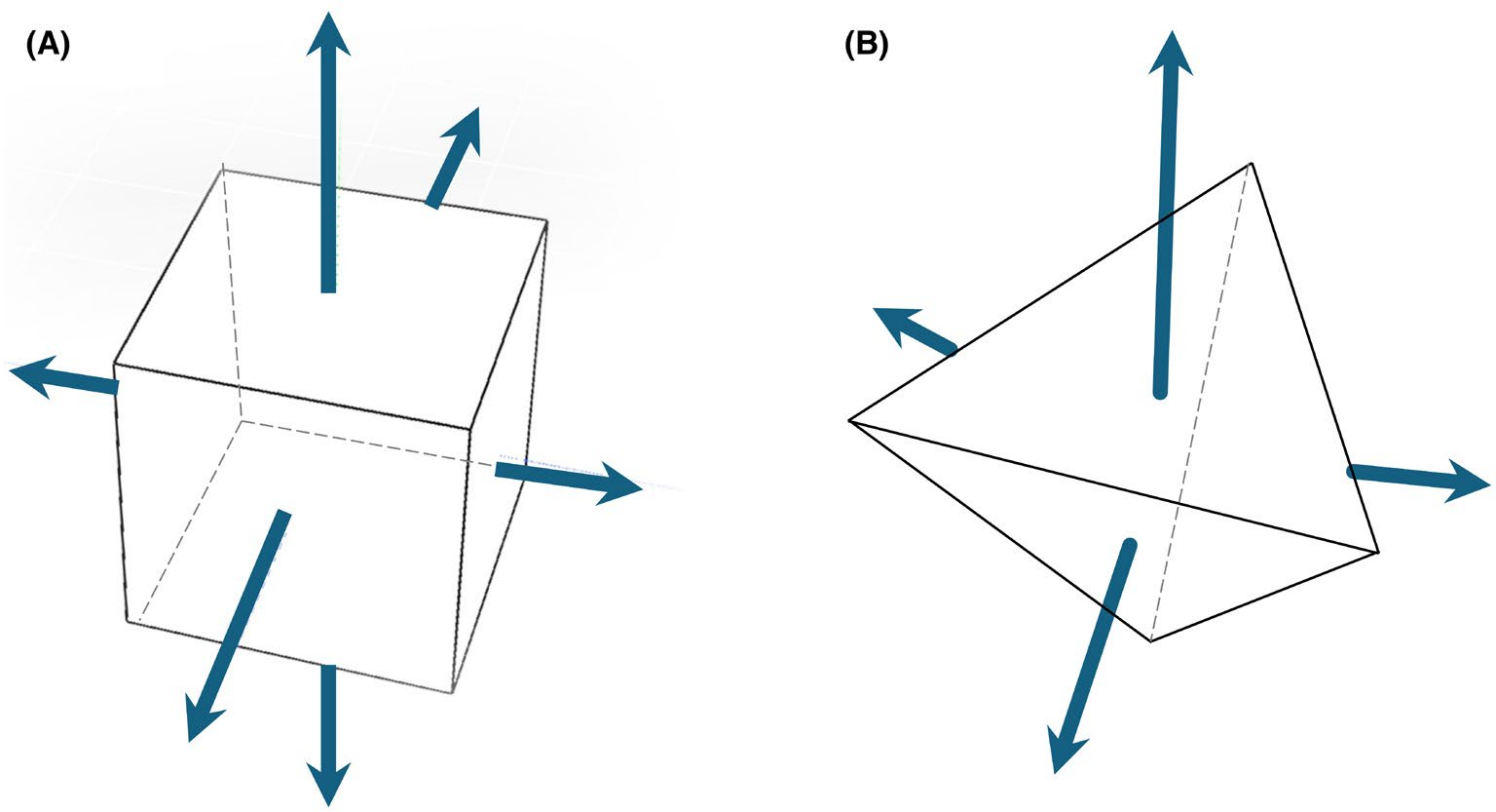
A diagram showing a cube with corresponding six measurements (A) and a tetrahedron with corresponding four measurements (B).

Rahn et al. used cubic irradiance as an approximation of fluence rate, but they found that it overestimated fluence rate compared to actinometry and the orientation of the cube had a large effect on the results.^15^ Bjorn proposed tetrahedral irradiance as an approximation of fluence rate, arguing that the cubic approximation can have a variation of up to 73% depending on the cube orientation.^18^ The tetrahedron approach has not been compared to actinometry in the past. Diagrams of both approaches are shown in Figure 1.

To address these gaps, we conducted two experiments. Experiment 1 was focused on fluence rate comparisons across two simulation tools, two measurement techniques using a radiometer, and spherical actinometry for UR and WR GUV systems. Additionally, Experiment 1 compared irradiance predictions from the two simulation tools to irradiance measurements for the WR system. In Experiment 2, irradiance predictions from the two simulation tools were further evaluated against measurements for UR and another WR GUV system.

The two experiments were conducted in two testing laboratories—Purdue University and Pacific Northwest National Laboratory (PNNL)—using one UR and one WR system in each laboratory. The selection of GUV systems was based on availability of products at the two locations and to include different products with different GUV wavelengths. Experiment 1 included LPM UR, whereas Experiment 2 included LED UR. The two experiments also used two different WR systems with different emitters and were from different vendors. We acknowledge that this decision makes the assessment of fluence rate and irradiance unique to each test site. This is further discussed in the limitations section.

## MATERIALS AND METHODS

### Experiment 1: Fluence rate and skin irradiance

#### Purdue experimental chamber

**FIGURE 2:**
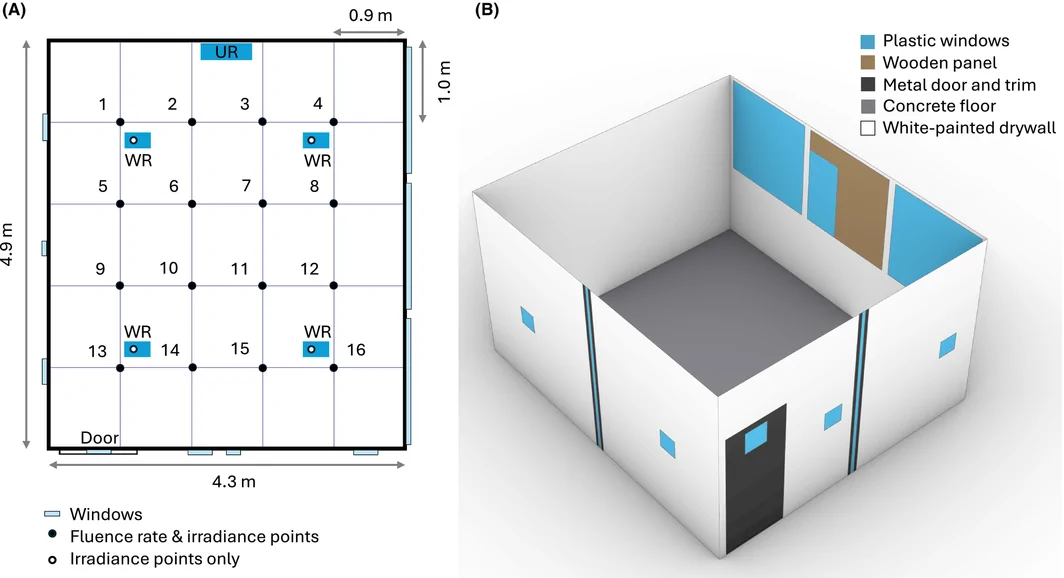
Plan view of the chamber including measurement grid and GUV system locations (A) and isometric view with material types (B).

The experimental chamber was 4.3 m × 4.9 m, with a ceiling height of 2.7 m. The floor was smooth concrete, and the walls and ceiling were white‐painted drywall. Other surfaces included plastic sheets that covered viewing windows from an adjacent space, a wooden panel and a metal door (Figure 2).

#### Testing scenarios

Measurements were collected under two GUV scenarios:

1. Four ceiling mounted WR GUV luminaires (Puro Protect 222), using KrCl* lamps with clear lenses, a rated input system power of 17 W, and peak wavelength of 222 nm. The centers of luminaires apertures were located 1.22 m from short walls and 1.07 m from long walls (Figure 2).
2. One wall‐mounted UR GUV luminaire (Aeromed Lexus L2.1) using an LPM lamp shielded by louvers with rated input system power of 25 W and peak wavelength of 254 nm. The bottom surface of the luminaire and the radiant aperture was at a height of 2.1 m, with luminaire center centered on a short wall and tilted 3° up from horizontal.

#### Measurements

For each GUV system, the same horizontal measurement grid centered in the room was used at two heights: 0.46 m and 0.91 m below the ceiling (referred to as upper and lower planes, respectively). The grid spacing was 0.9 m × 1.0 m. Fluence rate measurements were collected at each point of the grid at both heights using spherical actinometry and two measurement techniques (cubic and tetrahedron approximations). Horizontal irradiance measurements were also collected for the WR installation using the same grid and points directly underneath each luminaire (16 grid points and 4 points below each luminaire) at the heights of 1.3 m and 1.8 m above floor, corresponding to seated and standing head positions, respectively. All the irradiance measurements were collected with the full 180° field of view (FOV).

##### Chemical actinometry

The spherical actinometry measurements involved the use of 32 quartz spheres 1 cm in diameter (see Figure S1). The spheres were suspended from the ceiling of the chamber using monofilament fishing line and snap‐swivels to allow positioning at the two heights (0.46 m and 0.91 m below the ceiling) and 16 locations, as shown in Figure 2. Each quartz sphere contained an actinometer solution comprising 0.6 molar (M) KI, 0.1 M KIO_3_, and 0.01 M Na_2_B_4_O_7_*10 H_2_O, as described by Rahn.^16^

To account for the effects of thermal triiodide formation, a control sample was included. The control sample was prepared and analyzed at the same times as the exposed samples. Triiodide concentration in the control sample was subtracted from triiodide concentration measured in all UV‐exposed samples. Triiodide concentration in all actinometer solution samples was quantified by absorbance at 352 nm, which was measured with a UV‐Vis spectrophotometer (Shimadzu UV‐2700) using a 1.0 mm path length spectrophotometer cell.

Absorbed UV dose at each sphere location was calculated by the logic presented by Rahn et al.^15^ Specifically, the molar quantity of triiodide was calculated as the product of the molar concentration of triiodide and the volume of actinometer solution. The number of einsteins of radiation absorbed by the actinometer was calculated by dividing this value by the quantum yield for triiodide formation at the wavelength of exposure (see Table 1). The absorbed fluence (dose) was then calculated by dividing the number of einsteins absorbed by the cross‐sectional area of the sphere. The local fluence rate was calculated by dividing the absorbed fluence by the exposure time (see Table 1). The experiment with the WR GUV luminaires involved exposure of the filled quartz spheres for 3 h (10,800 s). The experiment with the UR GUV luminaire involved exposure of the quartz spheres for 25 min (1500 s).

**TABLE 1:** Parameter values applied in fluence rate calculations from spherical actinometry experiments.

Parameter	KrCl* (222 nm)	Low‐pressure mercury (254 nm)
Exposure time (*t*) (s)	10,800	1500
Quantum yield (Φ) $\left(\frac{\text{moles}}{\text{einstein}}\right)$	0.82^19^	0.695^20^

##### Irradiance

Irradiance measurements were collected using a calibrated radiometer (Gigahertz‐Optik X1‐UV‐3727‐5) with 1‐s integration time for both fluence rate approximations and skin irradiance. The calibration uncertainty was ±8.9% for 254 nm and ±26% for 222 nm. For fluence rate approximations, the radiometer was mounted on a gimbal that was controlled from a tablet that rotated the radiometer to the preset locations corresponding to the tetrahedron and cube sides, which included horizontal irradiance measurements as well.

##### Cubic approximation

By approximating an aerosol's volume to a cube, one can measure irradiance normal to each face and use these data to calculate fluence rate, as shown in Figure 1/left.^15^ For a typical detector with 180° FOV, measuring irradiance in directions 90° apart creates an overlap in the measurements and will lead to irradiation from some angles being counted twice. To reduce sensitivity in UV‐C measurements due to the orientation of the cube, another set of measurements was collected assuming a second cube rotated 45° around its vertical axis (Figure S2).

This method required collecting 10 irradiance measurements for each point to account for direct and reflected UV‐C: one with the detector aimed at zenith, one with the detector aimed at nadir, and eight with the detector aimed at the horizon, taken 45° apart by rotating the detector around the vertical axis. The 0° direction was pointing at the plan north wall where the UR GUV luminaire was located. The fluence rate was calculated by averaging the two sums of irradiance measurements for the sides of the first cube (0°, 90°, 180°, 270°) and the second cube (45°, 135°, 225°, 315°), and then adding the result to the zenith and nadir measurements.

(1) $$\begin{array}{c} \text{Fluence}\;\text{rate}=\frac{\left[\left(I_{0^{{}^{\circ}}}+I_{90^{{}^{\circ}}}+I_{180^{{}^{\circ}}}+I_{270^{{}^{\circ}}}\right)+\left(I_{45^{{}^{\circ}}}+I_{135^{{}^{\circ}}}+I_{225^{{}^{\circ}}}+I_{315^{{}^{\circ}}}\right)\right]}{2} \end{array}+I_{\text{Nadir}}+I_{\text{Zenith}}$$

##### Tetrahedron approximation

By approximating an aerosol's volume to a tetrahedron, one can measure irradiance normal to each face and use these data to calculate fluence rate, as shown in Figure 1, right.^15^ This method reduces the overlap in sensor fields of view compared to cubic irradiance measurements, thereby mitigating overestimation of fluence rate. To reduce sensitivity in UV‐C measurements due to the orientation of the tetrahedron, another set of measurements was collected assuming a second tetrahedron rotated 60° around its vertical axis (Figure S2).

This method required taking seven irradiance measurements for each point to account for direct and reflected UV‐C: one with the detector aimed at zenith, and six with the detector aimed 19.5° below the horizon, taken 60° apart by rotating the detector around the vertical axis. The 0° direction pointed at the plan north wall where the UR GUV luminaire was located. The fluence rate was calculated by averaging the two sums of tilted irradiance measurements for the sides of the first tetrahedron (0°, 120°, 240°) and the second tetrahedron (60°, 180°, 300°), and then adding the result to the zenith measurement:

(2) $$\begin{array}{c} \text{Fluence rate}=\frac{\left[\left(I_{0{}^{\circ}}+I_{120{}^{\circ}}+I_{240{}^{\circ}}\right)+\left(I_{60{}^{\circ}}+I_{180{}^{\circ}}+I_{300{}^{\circ}}\right)\right]}{2}+I_{\text{Zenith}} \end{array}$$

#### Simulations

Fluence rate and irradiance simulations were performed using two simulation software tools: Visual Lighting (version 2.12.0126) and Photopia (version 2022.0.0.11443). The dimensions of the simulation models matched the physical dimensions of the chamber. Plastic windows, a wooden panel, a metal door, and metal trim were included in the simulations. The reflectances assigned to the model surfaces are summarized in Table 2 and were based on previous studies using GUV with 222 nm or 254 nm wavelength.^21^, ^22^ There is very limited information on the specularity of materials in the GUV range, so all materials were assumed to be perfectly diffuse.

**TABLE 2:** Assumed UV‐C reflectance for Experiment 1 chamber surfaces.

Room surface	Material	Reflectance (%)	
		222 nm^21^	254 nm^22^
Floor	Concrete	6.1	14.9
Walls and ceiling	White painted drywall	7.8	6.7
Windows	Polycarbonate	8.2	8.2^a^
Panels and door	Stainless steel	28.9	24.5
Panel	Wood	8.6	4.7

^a^ No reflectance value was available for polycarbonate using a 254‐nm source, so it was estimated based on the reflectance value using a 222‐nm source.

The IES LM‐63 format data file for the UR luminaire was provided by the manufacturer and had the radiant power output of 652 mW. The IES file for the WR luminaire was provided by a third‐party laboratory that tested the luminaire at the beginning of its life, after 2 h of seasoning (i.e., burn‐in operation) and had the radiant power output of 99 mW. The radiant intensity distributions of the luminaires that were used in the simulations are shown in Figure S3. The light loss factors (LLFs) were assumed to be 1.0 for both luminaires, which included the UV portion of the output. Calculation grids for fluence rate and irradiance calculations matched what is shown in Figure 2.

##### Photopia

Photopia software uses the probabilistic ray‐tracing technique that models optical radiation as individual rays. Initial ray directions are determined based on IES files and corresponding probabilities. The user‐configurable parameters in Photopia include material types (diffuse, specular, or both) and reflectances (which can vary spectrally), sensor size, number of rays, reaction (i.e., ray‐surface interaction) count, IES files, and LLFs.

For fluence rate, the sensor size was chosen to be 1 cm to match the diameter of the actinometry spheres and for planar irradiance, the sensor size was chosen to be 1.92 cm to match the diameter of the acceptance area of the radiometer head. Sensitivity analysis found that 300 million rays, and a reaction count of 6 were sufficient for the results to converge in each scenario of this study.

##### Visual lighting

Visual lighting software uses the deterministic radiosity (i.e., radiative transfer) technique that calculates energy exchange between small patches of surfaces. This approach is based on the inverse‐square law, and all surface reflections are automatically assumed to be perfectly diffuse and spectrally uniform. The user‐configurable parameters in Visual® include material reflectances, IES files, and LLFs. Visual estimates fluence rate by calculating spherical irradiance on a representative volume at each calculation point.

### Experiment 2: Skin and eye irradiance

#### PNNL testing room

**FIGURE 3:**
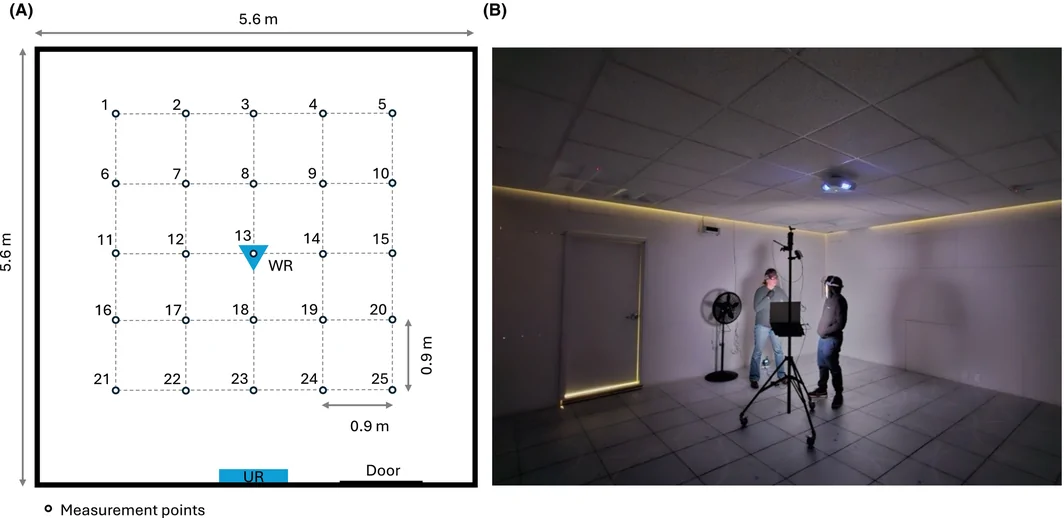
Testing room layout including measurement grid (A) and radiometric measurement setup (B).

The testing room used in Experiment 2 was 5.6 m × 5.6 m, with a ceiling height of 2.6 m (Figure 3). The floor was covered with gray plastic tiles, the walls were white‐painted drywall panels, and the ceiling was made of white fine‐fissured acoustic tiles. The room had one white‐painted door and no windows.

#### Testing scenarios

Measurements were collected for two GUV systems:

1. One ceiling mounted WR GUV luminaire (R‐Zero Vive). This luminaire uses three KrCl* lamps and has a rated input system power of 60 W and peak wavelength of 222 nm. The center of the luminaire and the measurement grid were located approximately in the center of the ceiling, offset from the center of the room 0.1 m plan northward and 0.03 m plan westward to allow for mounting to the ceiling grid (Figure 3). The luminaire's modules were tilted 45° up from nadir as recommended by manufacturer for the given ceiling height.
2. One wall‐mounted UR GUV luminaire (Excelitas Kepri). This luminaire uses a UV LED source with rated input system power of 30 W and peak wavelength of 270 nm; most of its output was in the UV‐C range, but 13% was in the UV‐B range (see Figure S4). The bottom of the luminaire bracket, which was 0.04 m above the bottom of the radiant aperture, was at a height of 2.1 m, with luminaire center approximately centered on the plan south wall, offset 0.03 m plan westward from the center of the room. The luminaire was tilted upward such that its aperture was 8° from vertical; integral safety controls would not allow it to operate with less tilt.

#### Irradiance measurements

The same radiometer from Experiment 1 was used. For each GUV system, a horizontal grid with 0.9 m × 0.9 m spacing was centered with respect to the WR luminaire (Figure 3). For the WR scenario, measurements were taken at two different heights: 1.3 m and 1.8 m above the floor (referred to as lower and upper planes), corresponding to seating and standing positions. For the UR scenario, measurements were only taken at the 1.8 m height per IES RP‐27.1‐22.^23^

The focus of this experiment was on skin and eye exposure, so UV‐C irradiance measurements were collected at each grid point in two ways: aimed at zenith with the full 180° FOV (to gauge skin exposure) and at horizon toward luminaire with the 80° FOV cone (to gauge eye exposure). The measurements were collected using a handheld radiometer (Gigahertz‐Optik X1‐UV‐3727‐5) with a 1‐s integration time.

#### Irradiance simulations

UV‐C irradiance simulations were performed using the same software approach as in Experiment 1. The reflectances assigned to the model surfaces are summarized in Table 3 and were based on previous studies using GUV with 222 nm or 254 nm wavelength.^21^, ^22^, ^24^

**TABLE 3:** Assumed UV‐C reflectance for Experiment 2 testing room surfaces.

Room surface	Material	Reflectance (%)	
		222 nm^21^	270 nm
Floor	Gray painted plastic tiles	7.5	7.5^a^
Walls	White painted drywall	7.8	6.7^22^
Ceiling	Fine fissured white tiles	19.6	27.4^b^

^a^ No reflectance value was available for gray paint for a 270‐nm or 254‐nm sources, so it was estimated based on the reflectance value for a 222‐nm source.

^b^ No reflectance value was available for fine fissured white tiles for a 270‐nm source, so it was estimated based on the reflectance value for a 254‐nm source (23).

The IES files for the UR and WR luminaires were generated by a third‐party laboratory that tested the luminaires after 500 h of use, which corresponded to their state at the time of measurement for this study; those same test units were used for the measurements in this experiment. The radiant power outputs of the IES files for WR and UR luminaires were 40 mW and 327 mW, respectively; their radiant intensity distributions used for the simulations are shown in Figure S5. The LLFs for both luminaires were set to 1.0.

Calculation grids for irradiance calculations matched what is shown in Figure 3. The 80° FOV cone was applied to eye irradiance simulations with a toggle switch in Visual software. In Photopia, the same assumptions and simulation parameters were used, and the 80° FOV cone was built and applied for the eye irradiance simulation sensors, with 80° FOV corresponding to the center of the sensor to match the geometry of the Gigahertz‐Optik cone that was used for the radiometric measurements.

### Statistical methods

All measurements and simulations conducted in this study intrinsically had errors associated with them that are discussed in limitations. For the purposes of comparison, actinometry measurements were chosen as a reference point for fluence rate comparisons, and radiometric measurements were chosen as a reference point for irradiance comparisons.

A graphical approach recommended by Bland and Altman^25^ and modified to account for a large range of fluence rate and irradiance values was used to evaluate agreement between measurements and simulations. Ratios of predicted to measured values and radiometric to actinometry measurements at each grid point were plotted on the vertical axis against their geometric mean on the horizontal axis, with logarithmic scales for both axes, as recommended by Rudnick et al. A regression line was also plotted for reference to highlight the data trends.

Median relative bias error (MRBE) was used to measure the systematic error between two sets of values; a smaller MRBE indicates smaller error. Equation 3 was used to calculate the relative bias error (RBE) for comparing fluence rates between radiometer‐based fluence rate (Test) and actinometry (Reference), and for comparing irradiance between simulations (Test) and radiometer measurements (Reference).

(3) $${\mathrm{RBE}}_{\text{Test}-\text{Reference}}=\left(\frac{{\text{Test}}_{i}-{\text{Reference}}_{i}}{{\text{Reference}}_{i}}\right)\times 100\%$$

Interquartile range (IQR) was used to measure the relative spread of relative bias error (RBE) around the median (Equation 4). A smaller IQR indicates smaller variation in RBE values.

(4) IQR=3rdQuartile−1stQuartile

Root mean square error (RMSE) was used to measure the absolute error for each scenario; a smaller RMSE indicates a smaller error. Equation 5 was used for comparing fluence rates between simulations (Test) and actinometry (Reference), for comparing fluence rates between measurement techniques using a radiometer (Test) and actinometry (Reference), and for comparing irradiance between simulations (Test) and radiometer‐based estimates (Reference).

(5) $${\text{RMSE}}_{\text{Test}-\text{Reference}}=\sqrt{\frac{\sum_{i=1}^{N}{\left({\text{Test}}_{i}-{\text{Reference}}_{i}\right)}^{2}}{N}}$$

## RESULTS

### Simulated fluence rate compared to actinometry

**FIGURE 4:**
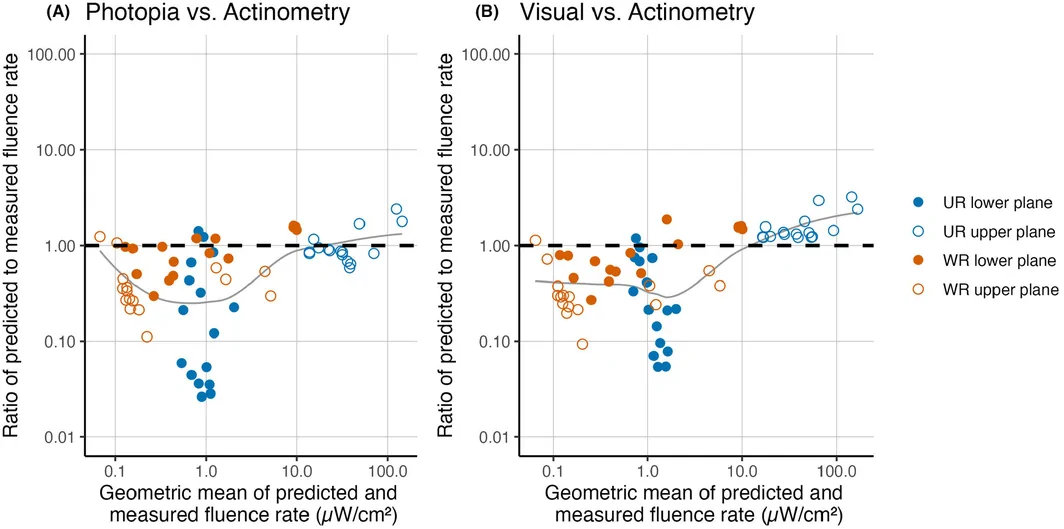
Modified Bland‐Altman plots of fluence rate comparing Photopia predictions (A) and Visual predictions (B) to actinometry measurements. The gray curved line represents locally estimated scatterplot smoothing (LOESS) regression fit to show the trend.

Figure 4 compares simulation predictions and actinometry measurements for fluence rate at individual measurement points, with tabulated values in Tables S2 and S3. The median of predicted to measured ratios was 0.65 and 0.68 for Photopia and Visual simulations, respectively, which suggests lower overall predicted fluence rate compared to actinometry. The lowest MRBE between simulations and actinometry was for WR lower plane and UR upper plane. Summary statistics comparing fluence rate between simulation predictions and actinometry measurements are shown in Table 4.

**TABLE 4:** Summary statistics comparing fluence rate between simulation predictions and actinometry measurements by plane height, including means, RMSE, MRBE, and IQR of RBE.

Statistics	Measurement plane	WR			UR		
		Photopia	Visual	Actinometry	Photopia	Visual	Actinometry
Mean (μW/cm^2^)	Upper	0.6	0.6	1.4	49.5	76.0	41.2
	Lower	3.4	3.4	2.4	0.5	0.6	3.0
	Both	2.0	2.0	1.9	25.0	38.3	22.1
RMSE (μW/cm^2^)	Upper	1.9	1.7	–	37.1	62.7	–
	Lower	2.1	2.1	–	3.3	3.2	–
	Both	2.0	1.9	–	26.3	44.4	–
MRBE (%)	Upper	−65	−70	–	−14	34	–
	Lower	−5	−21	–	−83	−78	–
	Both	−48	−52	–	−26	20	–
IQR of RBE (%)	Upper	20	14	–	18	38	–
	Lower	63	97	–	45	61	–
	Both	67	51	–	70	112	–

For the WR GUV and compared to actinometry, Photopia and Visual performed similarly predicting lower mean fluence rate for points in the upper plane, and higher average fluence rate for points in the lower plane. Considering all measurement points, both software predicted a mean fluence rate of 2 𝜇W/cm^2^ similar to 1.9 𝜇W/cm^2^ measured using actinometry.

For UR GUV, means of fluence rate predicted using Photopia or Visual were higher than actinometry in the upper plane and lower than actinometry in the lower plane. There was a large vertical spread in the ratio of simulated to measured fluence rate in the lower plane. Considering both measurement planes, mean fluence rate based on Photopia was slightly higher (25.0 𝜇W/cm^2^) than actinometry (22.1 𝜇W/cm^2^), whereas Visual was substantially higher by 73% (mean = 38.3 𝜇W/cm^2^).

### Fluence rate based on cubic and tetrahedron approximations compared to Actinometry

**FIGURE 5:**
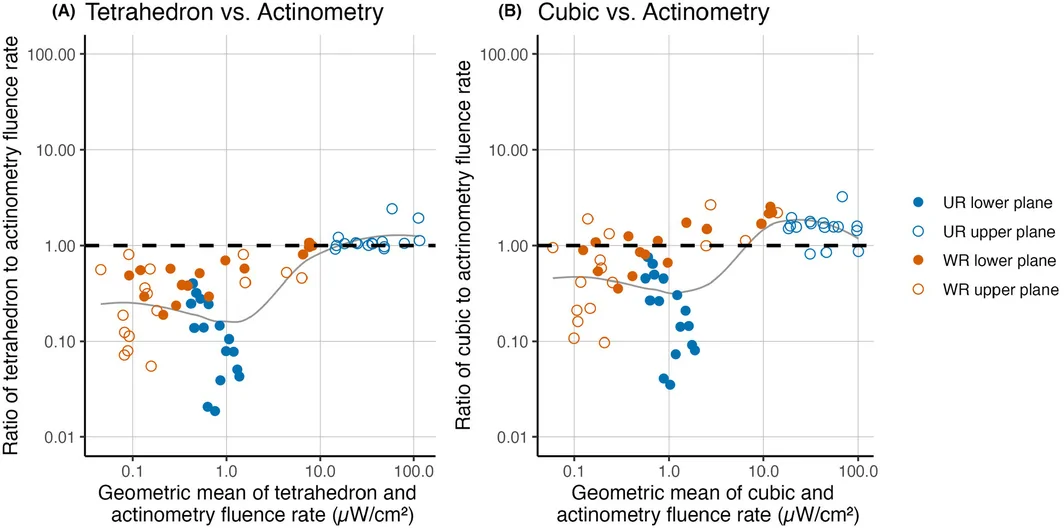
Modified Bland–Altman plots of fluence rate comparing a tetrahedron approximation technique (A) and a cubic approximation technique (B) to actinometry. The gray curved line represents locally estimated scatterplot smoothing (LOESS) regression fit to show the trend.

The comparisons between cubic and tetrahedron approximations and actinometry measurements for fluence rate for individual points are shown in Figure 5. Tabulated values are in supplementary materials Tables S2 and S3. Summary statistics comparing fluence rate between tetrahedron, cubic, and actinometry measurements are shown in Table 5. The agreement between tetrahedron and actinometry measurements improved as the fluence rate increased, except for the UR GUV scenario lower plane that did not follow that trend, similar to the results from simulation comparisons.

**TABLE 5:** Summary statistics comparing fluence rate between radiometric and actinometry measurements by plane height including means, RMSE, MRBE, and IQR of RBE.

Statistics	Measurement plane	WR			UR		
		Tetrahedron	Cube	Actinometry	Tetrahedron	Cube	Actinometry
Mean (μW/cm^2^)	Upper	0.7	2.2	1.4	50.9	60.4	41.2
	Lower	2.1	4.7	2.4	0.2	0.5	3.0
	Both	1.4	3.5	1.9	25.6	30.4	22.1
RMSE (μW/cm^2^)	Upper	1.5	2.9	–	23.3	29.7	–
	Lower	0.5	4.6	–	3.3	3.2	–
	Both	1.2	3.8	–	16.7	21.1	–
MRBE (%)	Upper	−66	−36	–	5	57	–
	Lower	−47	10	–	−88	−76	–
	Both	−57	−8	–	−34	−21	–
IQR of RBE (%)	Upper	41	96	–	10	21	–
	Lower	37	93	–	20	36	–
	Both	34	108	–	92	132	–

Mean fluence rate values using a tetrahedron approximation were lower compared to actinometry, except for the UR GUV scenario upper plane. In contrast, means of fluence rate using a cubic approximation were higher compared to actinometry except the UR GUV scenario lower plane. Considering both measurement planes, means of fluence rate based on a tetrahedron approximation were closer to the actinometry estimate and had lower RMSE than did the cubic measurements.

### Irradiance simulations compared to radiometric measurements

**FIGURE 6:**
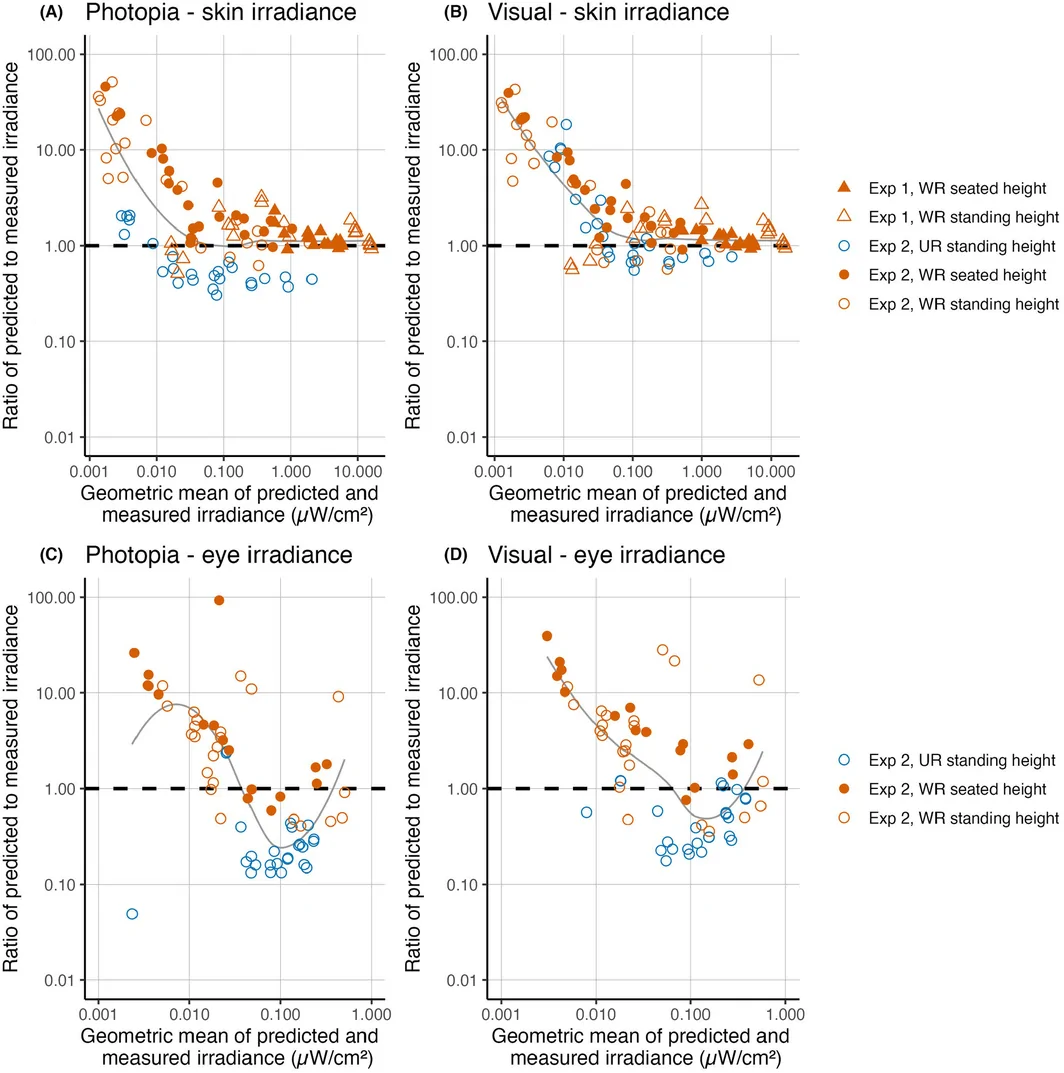
Modified Bland–Altman plots of irradiance comparing simulation predictions and radiometric measurements using Photopia (left column) and Visual (right column) for skin (top) and eye exposure (bottom). The gray curved line represents locally estimated scatterplot smoothing (LOESS) regression fit to show the trend.

The comparisons between simulation predictions and radiometric measurements of skin irradiance at individual points for both experiments are shown in the two upper graphs of Figure 6, with tabulated values shown in Tables S4–S6. RMSE, MRBE, and IQR comparisons between scenarios are summarized in Table 6; average irradiance values were not included, as only individual maximum irradiance values are typically used for photobiological safety prediction and assessment.

**TABLE 6:** Summary statistics comparing irradiance simulations of skin and eye exposure to radiometric measurements by plane height (seated 1.3 m and standing 1.8 m).

Measurement	Statistics	Measurement plane	WR (Exp 1)		WR (Exp 2)		UR (Exp 2)	
			Photopia	Visual	Photopia	Visual	Photopia	Visual
Skin exposure	RMSE (μW/cm^2^)	Seated	0.4	0.4	0.1	0.1	–	–
		Standing	1.8	1.8	0.1	0.1	0.4	0.2
		Overall	1.3	1.3	0.1	0.1	–	–
	MRBE (%)	Seated	7	11	107	141	–	–
		Standing	31	37	396	368	–50	0
		Overall	13	14	283	282	–	–
	IQR of RBE (%)	Seated	22	24	553	477	–	–
		Standing	64	76	1718	1633	33	223
		Overall	42	43	886	803	–	–
Eye exposure	RMSE (μW/cm^2^)	Seated	–	–	0.1	0.1	–	–
		Standing	–	–	0.3	0.4	0.2	0.2
		Overall	–	–	0.2	0.3	–	–
	MRBE (%)	Seated	–	–	289	241	–	–
		Standing	–	–	242	259	–80	–61
		Overall	–	–	242	259	–	–
	IQR of RBE (%)	Seated	–	–	1065	781	–	–
		Standing	–	–	482	505	14	51
		Overall	–	–	817	583	–	–

Radiometric measurements of skin irradiance ranged from 0 to approximately 17 𝜇W/cm^2^ (mean = 1.48 𝜇W/cm^2^). The median of predicted to measured values was 1.28 for Photopia simulations and 1.40 for Visual simulations.

Photopia simulations predicted lower skin irradiance compared to radiometric measurements for most points in the UR GUV scenario (MRBE = 50%). Both simulation tools predicted higher skin irradiance compared to radiometric measurements for most points in the WR GUV scenarios (MRBE = 13% to 283%). The agreement between simulations and measurements improved as skin irradiance increased for most scenarios and was closest for the points from Experiment 1 where the skin irradiance values were highest (MRBE = 13% to 14%).

The comparisons between simulations and measurements of eye irradiance (Experiment 2) are shown in the two lower graphs of Figure 6, with tabulated values shown in supplementary materials Tables S7 and S8. The median of predicted to measured values was 0.98 for Photopia simulations and 1.17 for Visual simulations. For UR GUV, simulations predicted lower eye irradiance compared to radiometric measurements for most points. For WR GUV, simulated values were generally higher than radiometric measurements.

## DISCUSSION

### Fluence rate simulations and measurements

Simulation predictions better aligned with actinometry measurements for the UR GUV upper plane and WR lower plane measurements. For UR GUV, it is worth noting that the lower plane was located below the main beam of the UR GUV luminaire and primarily received reflected irradiation (Figure S6), as opposed to mostly direct irradiation in the upper plane. The assumed reflectance values for the ceiling and walls could be a source of error in that scenario. Some of the points in the lower plane were also located along the luminaire's narrow beam edge, potentially sensitive to small differences in location. For WR GUV, the actinometry measurements in the upper plane could be more sensitive to interference from the rods supporting the quartz spheres when irradiated by ceiling‐mounted WR luminaires (Figure S1).

In practical applications, GUV effectiveness is typically evaluated assuming that the air is well mixed, making the mean fluence rate the most critical metric. The overall mean fluence rate showed close agreement between actinometry and both simulation software for WR GUV systems as well as between actinometry and Photopia for UR GUV systems.

The agreement between simulations and measurements tended to improve as the geometric mean values increased. A similar trend between measurements and simulations was found by Rudnick and colleagues where there was improved agreement between simulations and measurements as the fluence rate increased, up to a certain fluence rate range (19–51 μW/cm^2^), after which the agreement decreased.^6^

The trends between tetrahedron approximation of fluence rate and actinometry measurements were similar to those observed between simulations and actinometry measurements. The tetrahedron approximation tended to underestimate mean fluence rate compared to actinometry for UR lower plane and WR, with the agreement improving as fluence rate increased. For the upper plane in UR GUV where the fluence rate was >10 μW/cm^2^, the tetrahedron approximation aligned better with actinometry with a smaller difference in means, smaller RMSE, and MRBE, compared to a cubic approximation. The cubic approximation predicted higher mean fluence rate than actinometry for UR GUV upper plane (MRBE = 57%), consistent with a previous study by Rahn et al.^15^ In that study, the cubic fluence rate measurements tended to be higher than actinometry measurements, with an average from the cubic fluence rate measurements of 54 μW/cm^2^, compared to 42–43 μW/cm^2^ using actinometry.

### Irradiance simulations and measurements

When irradiance is simulated or measured at fixed points in a space for photobiological safety assessment, it is important to note that the underlying assumption is stationary occupants for an extended period, often an assumed 8‐h exposure. This is likely a conservative assumption for most cases except for scenarios where occupants spend most of their time in fixed positions and view directions, for example, office workers in a call center or patients in a hospital bed. A previous study found that occupants in a restaurant/club and a dental clinic only received approximately 10%–49% of the maximum measured stationary dose.^26^ For occupants who do not conform to the stationary assumption, their motion and varying view direction should be accounted for to make an exposure assessment.

For skin irradiance, the agreement between simulations and measurements improved as irradiance increased. For WR GUV, the closer agreement between simulations and radiometric measurements for skin irradiance values closer to 10 μW/cm^2^ is relevant and useful for WR GUV design to help eliminate hotspots for photobiological safety given the ACGIH time weighted skin irradiance limit of 7.4–8.8 μW/cm^2^ for an 8‐h exposure.^27^ For UR GUV, the ACGIH skin irradiance limit for an 8‐h exposure was ~0.4 μW/cm^2^, Visual simulations better matched radiometer measurements in the range 0.1–1 μW/cm^2^. Note that the ACGIH limits referenced here varied slightly depending on the specific spectrum.

Note that both software estimated skin irradiance to be above ACGIH limits for some points while the measurements were below these limits. In this case, relying solely on simulations might lead to designs that are overly conservative. The worse agreement at lower levels of irradiance below 0.1 μW/cm^2^ is less important for skin exposure.

Eye irradiance measurements were substantially lower (<1 μW/cm^2^) than skin irradiance. The agreement was generally worse and more dispersed than for skin irradiance. For the WR GUV system, the highest predicted or measured eye irradiance value was still lower than the 3–3.5 μW/cm^2^ time weighted average irradiance limit recommended by the ACGIH for an 8‐h eye exposure. Therefore, for this specific scenario a poor agreement between simulations and measurements is unlikely to be critical for photobiological safety predictions. More work is needed to assess the agreement between simulations and measurements for higher eye irradiance levels for WR GUV systems (in the range of 1–5 μW/cm^2^).

For the LED UR system, both software predicted lower eye irradiance compared to measurements with MRBE of −80% for Photopia and −61% for Visual. The ACGIH time weighted average irradiance limit for an 8‐h eye exposure was 0.12 μW/cm^2^. For eye irradiance values close to this limit, both simulation software generally predicted lower values than measured; this can be problematic when predicting photobiological safety for stationary occupants.

Since eye irradiance measurements used an 80° FOV cone, small deviations in location and orientation of the radiometer could have significant effects on the measurements compared to using a full 180° FOV, which could explain the poor agreement between simulations and measurements for eye irradiance.

Additional studies to replicate these findings and to pinpoint the sources of difference between simulations and measurements could be used to inform better procedures. Given that safety considerations are of utmost importance when designing GUV systems, the discrepancies between simulation predictions and measurements for eye and skin irradiance would need to be accounted for to prevent potential overexposure of stationary building occupants. For the specific scenarios and parameters studied here, a correction factor could be explored akin to safety factors used in other fields to provide a margin against risks. Modeling systems using the upper end estimates of room surface reflectances for safety predictions is another approach. Investigating additional applications, surface reflectances, geometries, and luminaires, and the impact that simulation parameters have on overall GUV system performance could confirm whether a correction factor approach could be feasible. Using dimmable or adjustable GUV luminaires that can be turned down or redirected in the field could be another approach to address discrepancies between simulated and measured performance.

## LIMITATIONS

The limitations discussed below are also sources of error for all measurements and simulations conducted in this study.

The two experiments evaluated fluence rate and irradiance in different rooms and used different GUV products that varied in their design, for example, source type, optics and louvers, and spatial distribution of emitted UV‐C. This created unique testing combinations that may have affected the results. For example, fluence rate was assessed under a LPM and a KrCl* product, whereas eye and skin exposure were evaluated using a LED source and another KrCl* product.

All IES files used for simulations have uncertainty associated with them. Room surface reflectance values used in the simulations were estimated based on previous studies and may not represent the specific surface materials in the chamber or the testing room. Due to the lack of data on surface UV‐C specular reflections, all surfaces were also assumed to be perfectly diffuse for all simulations; the polycarbonate windows and metal features in the Experiment 1 chamber may have some specular components in their reflections. Additionally, fluence rate and irradiance from upper‐room luminaires used in this study can be sensitive to small deviations in their tilt and positioning due to their relatively narrow beams, which can be a source of error.

One of the WR GUV luminaires used in Experiment 1 appeared to have a lower radiant output than would be expected from the available IES file, based on irradiance measurements directly underneath the luminaires. This is likely attributable to manufacturing tolerances, since measurements underneath the other luminaires agreed more closely with the IES file. Additionally, the IES file used for the WR luminaire in Experiment 1 was based on initial performance, because information on luminaire use (i.e., run time) was not available. For this specific WR luminaire, the output would be expected to increase over its first 500 h of operation based on third‐party lab testing.

There was uncertainty associated with the calibration of the radiometer, which was relatively high at 222 nm (±26%), compared to 254 nm (±8.9%). The radiometric measurements were more susceptible to noise at lower values, which could be a contributing factor to disagreements at lower fluence rates and irradiances.

The limited number of measurement locations for each system type was based on the time constraints and was not intended to comprehensively characterize these systems. Simulations allow for characterization over thousands of points and can provide a much more complete summary of the entire system performance reducing the overall dependence upon the sensitivity of limited measurement point placements.

The spherical actinometry experiments conducted in this work involved the KI/KIO_3_ actinometer. This actinometer solution is essentially opaque to UV‐C radiation. Therefore, virtually all photons that struck the actinometer solution were absorbed. Also, the quantum yield for this actinometer has been well characterized across the UV‐C spectrum.^20^, ^28^ However, photons that are incident on a quartz sphere containing this actinometer solution will encounter curved material interfaces (air:quartz and quartz:water) that include changes in refractive index. Therefore, reflection and refraction of incident radiation will take place at these material interfaces. These optical phenomena have not been accounted for in previously reported results from spherical actinometry experiments and were not accounted for in the experiments described herein, but may contribute to optical losses.

## Supporting information


Table S1.

## Data Availability

The data that support the findings of this study are available in the supplementary material of this article.
