# Supplementary material for: Evaluating tools for predicting and measuring radiometric performance of germicidal ultraviolet systems
Source: Photochem Photobiol. 2026 Feb 18;102(5):1123–37. doi: 10.1111/php.70080 (PMC13575708; doi:10.1111/php.70080)
Supplement: Supplementary file 1 [file PHP-102-1123-s001.docx]

**SUPPORTING MATERIALS**

**Radiometric measurements procedure**

1) Researchers wore personal protection equipment including an ANSI Z87.1+U6 face shield and covered all other skin with clothing upon entering the space or switching on GUV s.
2) Detector sensitivity to wavelengths outside the 200–280 nm UV-C wavelength range was observed during the pilot tests, so to minimize any impact on the measurements from out-of band-radiation, electric lighting was switched off and any available window blinds were closed.
3) An ozone logger was started to monitor ambient ozone levels.
4) The GUV luminaires were switched on and allowed to stabilize for approximately 30 minutes before fluence or exposure measurements started.
5) Grid points were marked on the floor.
6) The spectral irradiance distribution was measured, and corresponding irradiance limits were calculated.
7) Spot exposure measurements were collected to determine whether personal protection equipment was needed based on the exposure limits calculated in step 6 and an assumed eight-hour exposure duration.
8) Irradiance measurements were collected for fluence and exposure evaluation. Measurements were collected at 100% duty cycle.

The UV spectral irradiance distribution was measured for each make/model of GUV using a handheld-format spectroradiometer (International Light Technologies ILT960UV-RAA4).

**Photopia settings**

For fluence rate grids, Photopia places a recording sphere at the center of each cell, defined by rows and columns. The sphere size is determined by the distance between rows and columns or grid resolution. For planar irradiance grids, Photopia’s sensor size is determined by grid resolution the same way, except as planar rectangles instead of spheres. A 4 x 4 grid that is used as the basis for this study would generate very large spheres or sensors, which can be a problem, since fluence rate is defined using a very small sphere and planar irradiance is defined at a point in space, according to CIE. A very fine grid to ensure the sensor size is as small as possible could be a solution, but it takes several hours to run due to a large number of points. To reduce calculation time while ensuring reasonably small sensor sizes, individual calculation grids with 1 row and 1 column were created at each point of the grid.

**TABLES**

**Instrument calibration**

The X1-UV-3727-5 radiometer was calibrated by a third-party laboratory whose scope of accreditation covered the seven source/irradiance calibration scenarios specified in Table S1 below (which also presents the corresponding correction/uncertainty outcomes).

Table S1: Correction factors at different irradiance levels.

| **Source** | **Nominal irradiance**  **(µW/cm^2^)** | **Correction factor** | **Uncertainty** |
| --- | --- | --- | --- |
| 222 nm | 1 | 1.023 | ± 26% |
| 254 nm | 0.01 | 0.1957 | ± 8.9% |
|  | 0.1 | 0.5945 |  |
|  | 1 | 0.9856 |  |
|  | 10 | 1.005 |  |
|  | 100 | 0.9907 |  |
|  | 1,000 | 0.9822 |  |

Based on Table S1, irradiance values in the range 0.01–1 𝜇W/cm^2^ were corrected using multipliers calculated by fitting the corresponding values in Table S1 for the 254 nm source (Multiplier= 0.1715 $\times$ ln(Irradiance) + 0.9869). This correction was also applied to WR and UR LED irradiance given that the radiometer uses the same detector and applies a multiplier depending on the source 254nm, 222nm, and 270 nm. Values lower than 0.01 used the 0.01 correction factor.

The ILT960UV-RAA4 spectroradiometer used in this study was calibrated for spectral irradiance 200–500 nm by its manufacturer before the start of the study and its ISO/IEC 17025 scope of accreditation covered the calibration.

**Table S2:** Fluence rate [$\mu W/\mathrm{cm}^{2}$] at individual points from simulations and measurement techniques for the whole-room system.

| **Plane height** | **Points** | **Photopia** | **Visual** | **Tetrahedron** | **Cubic** | **Actinometry** |
| --- | --- | --- | --- | --- | --- | --- |
| **Upper plane (0.46 m from ceiling)** | 1 | 3.25 | 3.30 | 3.16 | 6.85 | 6.07 |
|  | 2 | 0.07 | 0.06 | 0.04 | 0.06 | 0.67 |
|  | 3 | 0.07 | 0.06 | 0.08 | 0.15 | 0.25 |
|  | 4 | 1.10 | 0.60 | 1.02 | 2.47 | 2.48 |
|  | 5 | 0.08 | 0.08 | 0.08 | 0.16 | 0.39 |
|  | 6 | 0.08 | 0.07 | 0.03 | 0.05 | 0.23 |
|  | 7 | 0.08 | 0.07 | 0.03 | 0.06 | 0.06 |
|  | 8 | 0.08 | 0.06 | 0.08 | 0.16 | 0.22 |
|  | 9 | 0.08 | 0.08 | 0.03 | 0.04 | 0.27 |
|  | 10 | 0.08 | 0.07 | 0.02 | 0.03 | 0.30 |
|  | 11 | 0.08 | 0.07 | 0.03 | 0.08 | 0.18 |
|  | 12 | 0.11 | 0.07 | 0.08 | 0.19 | 0.10 |
|  | 13 | 2.80 | 3.58 | 4.31 | 20.68 | 9.42 |
|  | 14 | 0.07 | 0.06 | 0.02 | 0.07 | 0.31 |
|  | 15 | 0.07 | 0.06 | 0.11 | 0.27 | 0.20 |
|  | 16 | 0.99 | 0.64 | 1.36 | 4.49 | 1.69 |
| **Lower plane (0.91 m from ceiling)** | 1 | 12.07 | 12.29 | 8.23 | 18.29 | 8.27 |
|  | 2 | 0.86 | 0.60 | 0.37 | 0.81 | 0.72 |
|  | 3 | 1.51 | 2.13 | 1.18 | 3.07 | 2.06 |
|  | 4 | 11.47 | 11.37 | 7.91 | 18.77 | 7.38 |
|  | 5 | 0.30 | 0.33 | 0.24 | 0.51 | 0.63 |
|  | 6 | 0.12 | 0.11 | 0.07 | 0.13 | 0.24 |
|  | 7 | 0.14 | 0.13 | 0.09 | 0.17 | 0.49 |
|  | 8 | 0.26 | 0.25 | 0.14 | 0.28 | 0.60 |
|  | 9 | 0.36 | 0.30 | 0.21 | 0.46 | 0.54 |
|  | 10 | 0.13 | 0.10 | 0.06 | 0.12 | 0.13 |
|  | 11 | 0.15 | 0.13 | 0.09 | 0.18 | 0.16 |
|  | 12 | 0.32 | 0.23 | 0.19 | 0.42 | 0.33 |
|  | 13 | 12.15 | 12.35 | 7.48 | 16.69 | 7.76 |
|  | 14 | 0.99 | 0.61 | 0.35 | 0.79 | 1.19 |
|  | 15 | 1.38 | 2.17 | 0.81 | 2.01 | 1.16 |
|  | 16 | 11.74 | 11.45 | 5.89 | 12.32 | 7.30 |

**Table S3:** Fluence rate [$\mu W/\mathrm{cm}^{2}$] at individual points from simulations and measurement techniques for the upper-room system.

| **Plane height** | **Points** | **Photopia** | **Visual** | **Actinometry** | **Tetrahedron** | **Cube** |
| --- | --- | --- | --- | --- | --- | --- |
| **Upper plane (0.46 m from ceiling)** | 1 | 29.2 | 61.9 | 50.01 | 46.29 | 42.37 |
|  | 2 | 193.9 | 259.4 | 80.67 | 155.49 | 115.91 |
|  | 3 | 193.4 | 258.9 | 108.20 | 122.10 | 93.76 |
|  | 4 | 29.3 | 61.6 | 34.48 | 36.50 | 28.11 |
|  | 5 | 31.3 | 59.9 | 49.01 | 47.64 | 76.80 |
|  | 6 | 63.5 | 111.3 | 37.70 | 91.05 | 121.99 |
|  | 7 | 63.8 | 111.2 | 77.30 | 81.48 | 122.65 |
|  | 8 | 29.9 | 59.8 | 43.81 | 48.16 | 68.68 |
|  | 9 | 21.2 | 32.1 | 23.32 | 24.92 | 41.47 |
|  | 10 | 28.4 | 43.2 | 35.48 | 36.98 | 55.17 |
|  | 11 | 28.5 | 43.1 | 32.65 | 32.79 | 55.96 |
|  | 12 | 21.6 | 32.1 | 24.47 | 25.62 | 41.54 |
|  | 13 | 12.7 | 18.4 | 14.91 | 14.91 | 22.46 |
|  | 14 | 16.9 | 22.2 | 17.82 | 18.65 | 27.78 |
|  | 15 | 16.5 | 22.2 | 14.15 | 17.27 | 27.59 |
|  | 16 | 12.5 | 18.4 | 15.14 | 13.88 | 24.32 |
| **Lower plane (0.91 m from ceiling)** | 1 | 0.14 | 0.30 | 5.51 | 0.10 | 0.19 |
|  | 2 | 0.15 | 0.47 | 3.29 | 0.35 | 0.68 |
|  | 3 | 0.13 | 0.47 | 2.22 | 0.32 | 0.67 |
|  | 4 | 0.16 | 0.31 | 4.36 | 0.09 | 0.18 |
|  | 5 | 0.26 | 0.41 | 1.22 | 0.17 | 0.33 |
|  | 6 | 0.20 | 0.45 | 5.81 | 0.29 | 0.53 |
|  | 7 | 0.19 | 0.36 | 6.65 | 0.28 | 0.54 |
|  | 8 | 0.23 | 0.42 | 4.36 | 0.17 | 0.32 |
|  | 9 | 0.56 | 0.63 | 0.84 | 0.21 | 0.38 |
|  | 10 | 0.43 | 0.74 | 3.52 | 0.28 | 0.50 |
|  | 11 | 0.43 | 0.68 | 0.99 | 0.28 | 0.49 |
|  | 12 | 0.49 | 0.63 | 1.53 | 0.21 | 0.40 |
|  | 13 | 0.98 | 0.82 | 0.69 | 0.28 | 0.52 |
|  | 14 | 1.10 | 0.96 | 1.30 | 0.32 | 0.59 |
|  | 15 | 0.97 | 0.93 | 4.28 | 0.33 | 0.62 |
|  | 16 | 1.04 | 0.81 | 0.84 | 0.27 | 0.54 |

**Table S4:** Skin irradiance [$\mu W/\mathrm{cm}^{2}$] at individual points from simulations and measurements for the whole-room system in Experiment 1.

| **Points** | **WR Exp 1** | | | | | |
| --- | --- | --- | --- | --- | --- | --- |
|  | **Seated height**  **(1.3 m above floor)** | | | **Standing height**  **(1.8 m above floor)** | | |
|  | **Photopia** | **Visual** | **Radiometry** | **Photopia** | **Visual** | **Radiometry** |
| 1 | 5.11 | 5.09 | 4.90 | 11.25 | 11.40 | 8.09 |
| 2 | 3.23 | 3.04 | 3.11 | 0.62 | 0.39 | 0.22 |
| 3 | 3.64 | 3.69 | 3.58 | 1.06 | 1.57 | 0.85 |
| 4 | 4.98 | 4.92 | 5.31 | 10.63 | 10.33 | 7.79 |
| 5 | 2.16 | 2.32 | 1.81 | 0.16 | 0.19 | 0.12 |
| 6 | 0.76 | 0.63 | 0.44 | 0.02 | 0.01 | 0.02 |
| 7 | 0.85 | 1.06 | 0.93 | 0.01 | 0.03 | 0.03 |
| 8 | 1.98 | 2.07 | 1.62 | 0.13 | 0.13 | 0.05 |
| 9 | 2.20 | 2.23 | 2.17 | 0.17 | 0.16 | 0.10 |
| 10 | 0.87 | 0.60 | 0.38 | 0.02 | 0.01 | 0.02 |
| 11 | 0.92 | 1.00 | 0.69 | 0.02 | 0.02 | 0.03 |
| 12 | 2.11 | 1.98 | 1.54 | 0.15 | 0.11 | 0.09 |
| 13 | 5.09 | 5.11 | 4.49 | 11.31 | 11.48 | 7.36 |
| 14 | 3.29 | 3.08 | 2.33 | 0.65 | 0.40 | 0.20 |
| 15 | 3.61 | 3.67 | 3.48 | 1.04 | 1.62 | 0.60 |
| 16 | 4.88 | 4.96 | 4.55 | 10.56 | 10.42 | 5.73 |
| 17 | 5.79 | 5.78 | 5.38 | 15.51 | 15.59 | 15.02 |
| 18 | 5.73 | 5.78 | 5.47 | 15.36 | 15.59 | 16.55 |
| 19 | 5.82 | 5.78 | 5.84 | 15.54 | 15.59 | 13.93 |
| 20 | 5.78 | 5.78 | 5.13 | 15.47 | 15.59 | 15.36 |

**Table S5:** Skin irradiance [$\mu W/\mathrm{cm}^{2}$] at individual points from simulations and measurements for the whole-room system in Experiment 2.

| **Points** | **WR Exp 2** | | | | | |
| --- | --- | --- | --- | --- | --- | --- |
|  | **Seated height**  **(1.3 m above floor)** | | | **Standing height**  **(1.8 m above floor)** | | |
|  | **Photopia** | **Visual** | **Radiometry** | **Photopia** | **Visual** | **Radiometry** |
| 1 | 0.01 | 0.01 | 0.00 | 0.00 | 0.00 | 0.00 |
| 2 | 0.03 | 0.07 | 0.03 | 0.01 | 0.01 | 0.00 |
| 3 | 0.47 | 0.46 | 0.33 | 0.11 | 0.10 | 0.14 |
| 4 | 0.04 | 0.08 | 0.03 | 0.01 | 0.01 | 0.00 |
| 5 | 0.01 | 0.01 | 0.00 | 0.00 | 0.00 | 0.00 |
| 6 | 0.01 | 0.01 | 0.00 | 0.01 | 0.01 | 0.00 |
| 7 | 0.03 | 0.03 | 0.01 | 0.03 | 0.03 | 0.01 |
| 8 | 1.28 | 1.25 | 0.85 | 1.95 | 1.79 | 1.84 |
| 9 | 0.04 | 0.04 | 0.00 | 0.03 | 0.03 | 0.00 |
| 10 | 0.01 | 0.01 | 0.00 | 0.01 | 0.01 | 0.00 |
| 11 | 0.04 | 0.04 | 0.01 | 0.01 | 0.01 | 0.00 |
| 12 | 0.17 | 0.17 | 0.04 | 0.21 | 0.26 | 0.12 |
| 13 | 0.12 | 0.12 | 0.06 | 0.38 | 0.37 | 0.27 |
| 14 | 0.22 | 0.21 | 0.11 | 0.23 | 0.30 | 0.22 |
| 15 | 0.05 | 0.04 | 0.02 | 0.02 | 0.01 | 0.00 |
| 16 | 0.23 | 0.19 | 0.18 | 0.04 | 0.03 | 0.03 |
| 17 | 0.53 | 0.50 | 0.55 | 0.26 | 0.24 | 0.42 |
| 18 | 0.04 | 0.03 | 0.00 | 0.05 | 0.05 | 0.01 |
| 19 | 0.67 | 0.64 | 0.37 | 0.37 | 0.34 | 0.36 |
| 20 | 0.28 | 0.23 | 0.15 | 0.04 | 0.03 | 0.05 |
| 21 | 0.04 | 0.04 | 0.03 | 0.00 | 0.00 | 0.00 |
| 22 | 0.03 | 0.02 | 0.00 | 0.01 | 0.01 | 0.00 |
| 23 | 0.01 | 0.01 | 0.00 | 0.01 | 0.01 | 0.00 |
| 24 | 0.04 | 0.03 | 0.01 | 0.01 | 0.01 | 0.00 |
| 25 | 0.05 | 0.05 | 0.03 | 0.01 | 0.01 | 0.00 |

**Table S6:** Skin irradiance [$\mu W/\mathrm{cm}^{2}$] at individual points from simulations and measurements for the upper-room system in Experiment 2.

| **Points** | **UR Exp 2** | | |
| --- | --- | --- | --- |
|  | **Standing height**  **(1.8 m above floor)** | | |
|  | **Photopia** | **Visual** | **Radiometry** |
| 1 | 0.00 | 0.02 | 0.00 |
| 2 | 0.01 | 0.03 | 0.00 |
| 3 | 0.01 | 0.05 | 0.00 |
| 4 | 0.01 | 0.03 | 0.00 |
| 5 | 0.00 | 0.02 | 0.00 |
| 6 | 0.01 | 0.03 | 0.01 |
| 7 | 0.01 | 0.04 | 0.03 |
| 8 | 0.02 | 0.06 | 0.02 |
| 9 | 0.01 | 0.04 | 0.02 |
| 10 | 0.01 | 0.03 | 0.02 |
| 11 | 0.02 | 0.04 | 0.05 |
| 12 | 0.04 | 0.08 | 0.12 |
| 13 | 0.05 | 0.11 | 0.10 |
| 14 | 0.04 | 0.08 | 0.14 |
| 15 | 0.02 | 0.04 | 0.05 |
| 16 | 0.06 | 0.09 | 0.13 |
| 17 | 0.16 | 0.27 | 0.42 |
| 18 | 0.27 | 0.46 | 0.61 |
| 19 | 0.16 | 0.27 | 0.40 |
| 20 | 0.06 | 0.09 | 0.11 |
| 21 | 0.10 | 0.17 | 0.15 |
| 22 | 0.57 | 1.01 | 1.21 |
| 23 | 1.37 | 2.37 | 3.09 |
| 24 | 0.56 | 1.03 | 1.50 |
| 25 | 0.10 | 0.17 | 0.17 |

**Table S7:** Eye irradiance [$\mu W/\mathrm{cm}^{2}$] at individual points from simulations and measurements for the whole-room system in Experiment 2. Point 13 was omitted, since it is impossible to aim the sensor at horizon toward luminaire right underneath the luminaire.

| **Points** | **WR Exp 2** | | | | | |
| --- | --- | --- | --- | --- | --- | --- |
|  | **Seated height**  **(1.3 m above floor)** | | | **Standing height**  **(1.8 m above floor)** | | |
|  | **Photopia** | **Visual** | **Radiometry** | **Photopia** | **Visual** | **Radiometry** |
| 1 | 0.01 | 0.02 | 0.00 | 0.02 | 0.02 | 0.00 |
| 2 | 0.04 | 0.12 | 0.05 | 0.02 | 0.03 | 0.02 |
| 3 | 0.43 | 0.70 | 0.24 | 0.24 | 0.26 | 0.53 |
| 4 | 0.05 | 0.14 | 0.05 | 0.02 | 0.03 | 0.01 |
| 5 | 0.01 | 0.02 | 0.00 | 0.02 | 0.02 | 0.00 |
| 6 | 0.01 | 0.02 | 0.00 | 0.02 | 0.03 | 0.01 |
| 7 | 0.01 | 0.00 | 0.00 | 0.04 | 0.05 | 0.01 |
| 8 | 0.04 | 0.00 | 0.00 | 1.31 | 1.95 | 0.14 |
| 9 | 0.01 | 0.00 | 0.00 | 0.04 | 0.06 | 0.01 |
| 10 | 0.01 | 0.02 | 0.00 | 0.03 | 0.03 | 0.00 |
| 11 | 0.04 | 0.06 | 0.01 | 0.03 | 0.03 | 0.01 |
| 12 | 0.01 | 0.00 | 0.00 | 0.14 | 0.27 | 0.01 |
| 13 | - | - | - | - | - | - |
| 14 | 0.01 | 0.00 | 0.00 | 0.16 | 0.31 | 0.01 |
| 15 | 0.04 | 0.07 | 0.02 | 0.03 | 0.04 | 0.01 |
| 16 | 0.27 | 0.33 | 0.24 | 0.10 | 0.09 | 0.20 |
| 17 | 0.20 | 0.00 | 0.00 | 0.34 | 0.45 | 0.68 |
| 18 | 0.00 | 0.00 | 0.00 | 0.03 | 0.05 | 0.00 |
| 19 | 0.26 | 0.00 | 0.00 | 0.48 | 0.63 | 0.53 |
| 20 | 0.31 | 0.40 | 0.19 | 0.11 | 0.09 | 0.26 |
| 21 | 0.06 | 0.08 | 0.10 | 0.02 | 0.02 | 0.03 |
| 22 | 0.03 | 0.04 | 0.01 | 0.02 | 0.02 | 0.01 |
| 23 | 0.01 | 0.02 | 0.00 | 0.03 | 0.03 | 0.01 |
| 24 | 0.04 | 0.05 | 0.01 | 0.02 | 0.02 | 0.01 |
| 25 | 0.09 | 0.11 | 0.11 | 0.02 | 0.02 | 0.02 |

**Table S8:** Eye irradiance [$\mu W/\mathrm{cm}^{2}$] at individual points from simulations and measurements for the upper-room system in Experiment 2.

| **Points** | **UR Exp 2** | | |
| --- | --- | --- | --- |
|  | **Standing height**  **(1.8 m above floor)** | | |
|  | **Photopia** | **Visual** | **Radiometry** |
| 1 | 0.02 | 0.02 | 0.10 |
| 2 | 0.02 | 0.03 | 0.11 |
| 3 | 0.02 | 0.03 | 0.06 |
| 4 | 0.02 | 0.03 | 0.13 |
| 5 | 0.02 | 0.02 | 0.13 |
| 6 | 0.03 | 0.05 | 0.19 |
| 7 | 0.04 | 0.06 | 0.28 |
| 8 | 0.04 | 0.07 | 0.18 |
| 9 | 0.04 | 0.06 | 0.23 |
| 10 | 0.03 | 0.04 | 0.21 |
| 11 | 0.05 | 0.09 | 0.28 |
| 12 | 0.07 | 0.15 | 0.46 |
| 13 | 0.09 | 0.18 | 0.35 |
| 14 | 0.07 | 0.15 | 0.50 |
| 15 | 0.05 | 0.09 | 0.28 |
| 16 | 0.08 | 0.17 | 0.32 |
| 17 | 0.12 | 0.34 | 0.43 |
| 18 | 0.13 | 0.31 | 0.31 |
| 19 | 0.13 | 0.34 | 0.43 |
| 20 | 0.08 | 0.18 | 0.32 |
| 21 | 0.09 | 0.23 | 0.20 |
| 22 | 0.04 | 0.02 | 0.02 |
| 23 | 0.00 | 0.01 | 0.01 |
| 24 | 0.04 | 0.02 | 0.02 |
| 25 | 0.08 | 0.23 | 0.21 |

**FIGURES**

**Figure S1.** Chemical actinometry sphere suspended from the ceiling.

**Figure S2.** A top view diagram of two intersecting cubes, and the corresponding eight side measurements (left); a top view diagram of intersecting tetrahedrons, and the corresponding six side measurements (right).

**Figure S3.** Polar plots of relative UV-C radiant intensity used for GUV luminaires in Experiment 1. WR luminaire plot is shown on the left, and UR luminaire plot is shown on the right.

**Figure S4.** Spectral irradiance distribution for the upper-room LED luminaire used in Experiment 2.

**Figure S5.** Polar plots of relative UV-C radiant intensity used for GUV luminaires in Experiment 2. WR luminaire plot for one of its lamps is shown on the left, and UR luminaire plot is shown on the right.

**Figure S6.** Section view of the Purdue IAQ Chamber UR scenario showing the luminaire’s radiant intensity distribution web and measurement/simulation grid points.
